# Effect of tracheal suctioning on aspiration past the tracheal tube cuff in mechanically ventilated patients

**DOI:** 10.1186/2110-5820-2-45

**Published:** 2012-11-07

**Authors:** Pascal Beuret, Bénédicte Philippon, Xavier Fabre, Mahmoud Kaaki

**Affiliations:** 1 Intensive Care Unit, Centre Hospitalier Roanne, 28 rue de Charlieu, 42328, Roanne, France

**Keywords:** Tube cuff, Aspiration, Suctioning maneuver

## Abstract

**Background:**

This clinical study evaluated the effect of a suctioning maneuver on aspiration past the cuff during mechanical ventilation.

**Methods:**

Patients intubated for less than 48 hours with a PVC-cuffed tracheal tube, under mechanical ventilation with a PEEP ≥5 cm H_2_O and under continuous sedation, were included in the study. At baseline the cuff pressure was set at 30 cm H_2_O. Then 0.5ml of blue dye diluted with 3 ml of saline was instilled into the subglottic space just above the cuff. Tracheal suctioning was performed using a 16-French suction catheter with a suction pressure of – 400 mbar. A fiberoptic bronchoscopy was performed before and after the suctioning maneuver, looking for the presence of blue dye in the folds within the cuff wall or in the trachea under the cuff. The sealing of the cuff was defined by the absence of leakage of blue dye either in the cuff wall or in the trachea under the cuff.

**Results:**

Twenty-five patients were included. The size of the tracheal tube was 7-mm ID for 5 patients, 7.5-mm ID for 16 patients, and 8-mm ID for four patients. Blue dye was never seen in the trachea under the cuff before suctioning and only in one patient (4%) after the suctioning maneuver. Blue dye was observed in the folds within the cuff wall in 6 of 25 patients before suctioning and 11 of 25 after (*p* = 0.063). Overall, the incidence of sealing of the cuff was 76% before suctioning and 56% after (*p* = 0.073).

**Conclusions:**

In patients intubated with a PVC-cuffed tracheal tube and under mechanical ventilation with PEEP ≥5 cm H2O and a cuff pressure set at 30 cm H2O, a single tracheal suctioning maneuver did not increase the risk of aspiration in the trachea under the cuff.

**Trial registration:**

ClinicalTrials.gov, number NCT01170156

## Background

The leakage of oropharyngeal secretions past high-volume low-pressure tracheal tube cuffs is usually considered a major risk factor for bacterial tracheal colonization and subsequent development of ventilator-associated pneumonia [1,2]. It has been demonstrated in a benchtop model that the rate of leakage around the cuff is related to the pressure differential across the cuff, namely the difference between the pressure of the subglottic fluid above the cuff and the tracheal pressure under the cuff [3]. Positive end-expiratory pressure (PEEP) improves the sealing around the cuff toward fluid leakage [4-6]. However, this preventive effect of PEEP may be compromised during prolonged mechanical ventilation by tracheal suctioning maneuver, which decreases tracheal pressure and enhances fluid leakage in vitro [3,7,8]. This clinical pilot study evaluated the effect of a suctioning maneuver on aspiration past the cuff during conventional mechanical ventilation.

## Methods

### Patients

The study was approved by the Committee for protection of humans in biomedical research Sud Est I. During the time of the study, the patients who needed invasive ventilation in the ICU were orally intubated with the same type of endotracheal tube (HI-LO Evac, Covidien, Elancourt, France), which is equipped with an additional lumen for access to the subglottic space above the polyvinyl chloride (PVC) cuff. The tracheal tube size was chosen by the physician in charge of the patient, usually 7-mm ID for women and 7.5- or 8-mm ID for men. Patients older than age 18 years were eligible for the study if they were intubated for less than 48 hours, under mechanical ventilation with a PEEP ≥5 cm H_2_O and under continuous sedation. Exclusion criteria were a known allergy to dye and hemodynamic failure. As approved by the Committee for protection of humans in biomedical research SudEst I, patients were included according to an emergency procedure. A deferred informed consent was asked from the patient’s surrogate as soon as possible. As he/she recovered consciousness, a deferred informed consent was asked from the patient. If the patient or his/her next of kin refused to consent, patient’s data were not entered into analysis.

The patients were studied in the semirecumbent position. At baseline, the cuff pressure was set at 30 cm H_2_O with a cuff inflator (Mallinckrodt Laboratories, Athlone, Ireland). Then, 0.5 ml of blue dye diluted with 3 ml of saline was instilled into the subglottic space just above the cuff. Tracheal suctioning was performed without disconnection of the ventilator, using a semiclosed system via a swivel adapter (Mallinckrodt DAR, Mirandola, Italy). The suctioning procedure was applied consistently throughout the study and was performed by the same investigator. The size of the suction catheter was 16-French, and its length was 47 cm (Vygon, Ecouen, France). The extent of negative pressure was 400 mbar, and the suctioning maneuver was standardized: the suction catheter was introduced in the tracheal tube with his total length, the suction negative pressure was then applied, the suction catheter maintained in distal position during 2 seconds and then withdrawn, and the suction negative pressure was applied for a whole duration of 10 seconds. A fiberoptic bronchoscopy (Pentax FI-16BS external diameter 5.2 mm) was performed before and immediately after the suctioning maneuver. The bronchoscope was advanced beyond the tracheal tube through the swivel adapter, looking for the presence of blue dye in the folds within the cuff wall or in the trachea caudal to the tube’s tip.

### Data collection

The following characteristics of each patient were recorded before the suctioning maneuver: age, sex, height, tracheal tube size, time from intubation, value of Ramsay score [9], mode of mechanical ventilation, the level of set tidal volume, respiratory rate and PEEP,and peak inspiratory pressure. The main evaluation criterion was the sealing of the cuff, defined by the absence of leakage of blue dye either in the cuff wall or in the trachea under the cuff.

### Statistical analysis

There was no clinical data reporting the effect of tracheal suctioning maneuver on aspiration. It was therefore a pilot study, and we decided arbitrarily to enroll 25 patients. Incidence rates were compared before and after tracheal suctioning maneuver using McNemar test. Numeric data were expressed as median (25th – 75th percentile). *P*<0.05 was considered statistically significant.

## Results

Twenty-five patients were included in the study; 22 of them were admitted in the ICU for a medical reason and 3 after an emergency surgery. Table 1 displays the characteristics of the patient population at baseline. They were intubated for a median duration of 18 hours (range, 11–31). The tracheal tube size was 7-mm ID for 5 patients, 7.5-mm ID for 16 patients, and 8-mm ID for 4 patients. The median ratio of suction catheter outer diameter to tracheal tube internal diameter was 0.7 (range, 0.7-0.7). All of the patients were under continuous sedation with midazolam and analgesia with fentanyl. Nine patients were paralyzed with continuous infusion of cisatracurium. The median Ramsay score of the patients who were not paralyzed was 5 (range, 5–6). All of the patients were ventilated in volume-controlled mode. The median level of PEEP was 5 cm H_2_O (5–5), peak inspiratory pressure 26 cm H_2_O (23–30), and inspiratory flow 26 l/min (22–30).

**Table 1:** Characteristics of the patient population

**Variable**	**Total n = 25**
Age (yr)	66 (55–76)
Sex (M/F)	13/12
Height (cm)	168 (160–172)
Internal diameter of tracheal tube (mm)	7.5 (7.5-7.5)
Ratio of suction catheter outer diameter to tracheal tube internal diameter	0.7 (0.7-0.7)
Previous duration of intubation (hr)	18 (11–31)
Ramsay score	6 (5–6)
Neuromuscular blockers use	9 (36)
Tidal volume (ml)	460 (380–530)
Respiratory rate (/min)	18 (17–22)
PEEP level (cm H_2_O)	5 (5–5)
Inspiratory flow (l/min)	26 (22–30)
Peak inspiratory pressure (cm H2O)	26 (23–30)

Data are number (%) or median (25th-75th percentile) values.

Before suctioning, the bronchoscopy depicted blue dye around the tube just above the cuff in all patients. Blue dye was observed in the folds within the cuff wall in 6 of 25 patients and never in the trachea under the cuff. After the suctioning maneuver, blue dye was observed in the folds within the cuff wall in 11 of 25 patients (*p* = 0.063) and only in one patient in the trachea under the cuff. Overall, the incidence of sealing of the cuff was 76% before suctioning and 56% after (*p* = 0.073).

## Discussion

In this study,the sealing of the tracheal tube cuff was not significantly altered by tracheal suctioning maneuver. High-volume low-pressure PVC cuffs have a diameter 1.5-2 times the diameter of the average adult trachea when fully inflated. Therefore, when these cuffs are inflated in a trachea to achieve a clinical seal, the excess material folds over itself and longitudinal channels appear in the cuff wall where subglottic secretions might leak to the lower airways. Numerous studies have shown frequent leakage of subglottic secretions past high-volume low-pressure tracheal tube cuffs, either in patients undergoing general anaesthesia [10-12] or in critically ill patients under mechanical ventilation [11,13,14], but the level of PEEP was never controlled in these studies. It has been recently demonstrated that PEEP is a critical factor for the prevention of leakage [4-6]. In the two clinical studies where PEEP was strictly set at ≥5 cm H2O, the incidence of leakage in the trachea under the cuff was low: 10% after 5 hours [6], and 0% after four hours in another study where the cuff pressure was checked every hour and reset at 30 cm H_2_O if needed [15]. However, in these two last studies, tracheal suctioning was not performed during the study period. In vitro tracheal suctioning maneuver, by decreasing tracheal pressure, induced a constant fluid leakage past the cuff, when performed either at a suction pressure of −200 cm H_2_O with a closed suction system [8], or at −400 mbar with a 16-French catheter suction with a semiclosed circuit [7]. For the purpose of this clinical study, we choose this latter size of suction catheter and extent of negative pressure, because it was our clinical practice.

Yet, in this clinical study the sealing of the cuff was not significantly altered by the suctioning maneuver. The incidence of leakage of blue dye in the folds within the cuff wall was doubled after suctioning, questioning the power of this study to detect a significant difference in leakage rate. However, the crucial issue, regarding the risk of bronchial colonization and development of ventilator-associated pneumonia, is the leakage of subglottic secretions in the lower airways under the cuff [16], which was only observed in one patient. To detect aspiration,we used blue dye as marker of subglottic secretions and bronchoscopic evaluation of leakage; this method is recognized as a reference diagnostic test and it is the sole direct method linking subglottic secretions and trachea under the cuff [16]. Several factors may explain why fluid leakage in the trachea under the cuff was constant after a suctioning maneuver in vitro but occurred rarely in this clinical study. First, in vitro fluid leakage was simulated by saline colored with blue dye. In clinical practice, subglottic secretions that pool above the cuff consist of saliva, whose viscosity may alter the pattern of leakage. Second, the plastic trachea used in vitro was connected to a test lung with a low compliance; the pressure change applied down the endotracheal tube during suctioning was then immediately transmitted around the cuff. In clinical practice, the fall of tracheal pressure results first in lung volume loss before transmitting to the cuff. Third, the inspiratory flow from the ventilator in the clinical study (median value 26 l/min) was higher than the one of in vitro study (12 l/min). Now the degree of negative airway pressure generated by suctioning depends on the balance between the inspiratory flow from the ventilator and the suction flow [17,18]. At a constant suction flow, the lower the inspiratory flow, the lower the negative pressure in the trachea.

Our study has some limitations. First, we did not control for factors that have been shown to influence leakage, such as the level of PEEP [4,5] and the tracheal tube size [4]. Second, the cuff pressure was set at 30 cm H_2_O just before the suctioning maneuver to study the effect of suctioning per se. This does not reflect the clinical practice, where the cuff pressure is usually checked every 8 hours;one study showed that patients intubated with PVC-cuffed tracheal tubes spent 26% of recording time at a cuff pressure below 20 cm H_2_O [19]. Moreover, we are unaware of the pattern of leakage in the hours following the suctioning maneuver and we did not evaluate the effect of repeated periodic suctioning maneuvers. Finally, the suction procedure we used was the worst condition favoring leakage and does not comply with recent guidelines, which recommend a negative pressure <150 mmHg and a ratio of suction catheter outer diameter to tracheal tube internal diameter <0.5, with the objective to limit the fall of intratracheal pressure [20]. The higher the suction pressure, the greater the rate of leakage will be [7]; also using a suction catheter with a large outer diameter increases endotracheal tube resistance and aspirated gas is not rapidly replaced by the inspiratory flow from the ventilator, thus increasing the fall of intratracheal pressure.

## Conclusions

In patients intubated with a PVC-cuffed tracheal tube cuff, in the conditions of the study (PEEP ≥5 cm H_2_O, cuff pressure set at 30 cm H_2_O), a single tracheal suctioning maneuver did not increase the risk of aspiration in the trachea under the cuff.

## Competing interests

The authors declare they have no competing interests.

## Authors’ contributions

PB designed the study, collected the data, performed the analysis, and drafted the manuscript. BP, XF, and MK recruited the patients for the study. All authors have read and approved the manuscript.
